# Effect of Mitral Regurgitation on Systemic Coagulation Activity in Rheumatic Heart Disease as Assessed by D-dimer Levels

**DOI:** 10.7759/cureus.17839

**Published:** 2021-09-09

**Authors:** Abhishek Goyal, Puneet Aggarwal, Abhinav Shrivastava, Bhagya Narayan Pandit, Saibal Mukhopadhyay, Jamal Yusuf, Vijay K Trehan

**Affiliations:** 1 Cardiology, Dayanand Medical College and Hospital, Ludhiana, IND; 2 Cardiology, Atal Bihari Vajpayee Institute of Medical Sciences (ABVIMS) and Dr. Ram Manohar Lohia (RML) Hospital, New Delhi, IND; 3 Cardiology, GB Pant Institute of Post Graduate Medical Education and Research, New Delhi, IND

**Keywords:** left atrial thrombus, coagulation, mitral valve regurgitation, mitral valve stenosis, rheumatic heart disease

## Abstract

Introduction

Atrial fibrillation and mitral stenosis, especially in combination, increase the risk of left atrial thrombus formation and systemic embolization. However, whether severe mitral regurgitation (MR) improves systemic hypercoagulable state in these patients is unclear. remains unclear. The study aims to study the impact of severe MR on systemic coagulation by the use of D-dimer levels.

Methods

It was a prospective, cross-sectional study done on 400 subjects consisting of 350 cases and 50 controls. The cases were divided into seven groups on basis of valvular pathology, rhythm, and presence of a clot. The D-dimer level was compared in all the subgroups.

Result

The mean age of the study population was 32.32±7.30 years with a 48% male population. The highest level of D-dimer was found in patients with thrombus (1.71 ± 1.74 µg/ml). Patients with mitral stenosis had significantly higher plasma D-dimer levels than the control group (p <0.001) while regardless of rhythm, patients with MR had a D-dimer level similar to the control group in sinus rhythm.

Conclusion

Severe MR reduces plasma D-dimer levels to control levels reflecting the protective effect against thrombus formation and systemic embolization.

## Introduction

Rheumatic heart disease (RHD) continues to be a major health problem in India and many other developing countries. Mitral stenosis (MS) and atrial fibrillation (AF) are dreaded complications of RHD. Both AF and MS cause stagnation of blood in the left atrium (LA), causing the Rouleaux formation of red blood cells, which is observed as echocardiographic ‘spontaneous echo contrast’ (SEC) or left atrial thrombus increasing the risk of systemic embolization [1-3]. The blood flow and endoluminal shear stresses must probably be below a critical value for intracardiac SEC and thrombus formation to occur.

It has been hypothesized that severe mitral regurgitation (MR) might be protective against LA thrombus formation [4-6]. This effect has been linked to high flow jet into LA and related high shear stress, which prevents stasis of blood into the left atrium and thus may prevent left atrial SEC and/or thrombus formation [5-11]. However, the impact of severe MR on the need for anticoagulation remains unclear in patients with MS and/or AF, as the coagulation cascade is likely activated in both disorders, resulting in a prothrombotic state [1,12-13].

Raised plasma D-dimer level is a marker of increased systemic coagulation and an active fibrinolytic system [14-16]. Since D-dimer levels are increased in the plasma of patients with AF, their levels can be measured to assess local intra-atrial hemodynamics and clot risk [1,12,17-23].

The present study aims to see the impact of severe rheumatic MR on systemic coagulation activity, as measured by D-dimer levels in patients with rheumatic mitral valve disease. It is hypothesized that severe MR would improve the systemic hypercoagulable state and reduce the coagulation profile to normal.

## Materials and methods

It was a prospective case-control, cross-sectional study. The study population had a total of 400 subjects consisting of 350 cases and 50 controls (age group 18-70 years) divided into eight subgroups (Table 1). Cases included patients diagnosed with rheumatic mitral valve disease on transthoracic echocardiography as per the criteria defined by World Heart Federation [24]. The cases were allocated to seven groups according to their rhythm, i.e. atrial fibrillation (AF) or normal sinus rhythm (NSR), valvular pathology (MS/MR/MS with MR), and the presence or absence of left atrium or left atrial appendage (LAA) thrombus. An equal number of participants (50 patients) in each group were recruited. Fifty healthy controls within the same age group and without any specific cardiac disease history served as the control group.

**Table 1:** Subgroups divided as per rhythm, valvular involvement, and presence of thrombus MS- mitral Stenosis; MR- mitral valve regurgitation; LA- left atrium; LAA- left atrial appendage; NSR- normal sinus rhythm; AF- atrial fibrillation

STUDY SUBGROUPS (N = 50 in each subgroup)	
1	Control
PATIENTS WITHOUT LA/LAA CLOT	
2	MS with NSR
3	MS with AF
4	MR with NSR
5	MR with AF
6	MS with MR with NSR
7	MS with MR with AF
PATIENTS WITH LA/LAA CLOT	
8	MS and/or MR with clot in LA/LAA

Patients over the age of 70, patients who had undergone surgery or had an acute coronary syndrome in the previous 12 weeks, patients on anticoagulation, patients with a history of stroke or systemic thromboembolism, deep vein thrombosis, patients with moderate or severe aortic valve disease, patients with left ventricular systolic failure with an ejection fraction of less than 40%, patients with an acute infection and patients with end-stage liver disease were all excluded from the study.

The study was done at a tertiary care center in India and well-informed written consent was taken from all study participants. Approval from the local institutional ethical committee of Maulana Azad Medical college was obtained prior to the study, with approval code number 124, and the study followed the code of declaration of Helsinki.

Eligible participants underwent complete clinical assessment and baseline laboratory tests (hemogram, erythrocyte sedimentation rate, renal function tests, liver function tests, antistreptolysin O (ASO) titers, and D-dimer level). Trans-thoracic echocardiography was performed to evaluate valvular involvement in the study group and to exclude cardiac disease in control subjects. Rheumatic heart valve disease was diagnosed as per the World Heart Federation criteria [24] and severity was assessed as per the American College of Cardiology (ACC)/American Heart Association (AHA) classification [25]. Patients with severe MS (mitral valve area <1.5 cm^2^) with concomitant progressive (or none) MR were classified as the MS subgroup, and patients with severe MR with mitral valve area >1.5 cm^2^ as the MR subgroup. Patients with mitral valve area <1.5 cm^2^ and severe MR were classified as the MS with MR subgroup. The D-dimer level was compared in all the subgroups.

Statistical analysis

Data were expressed as frequency (%) for qualitative variables. Quantitative variables were presented as mean ± SD for normal distribution (Kolmogorov-Smirnov goodness-of-fit test) and median + range for skewed distribution. If variables followed a normal distribution, differences between multiple groups were analyzed using a one-way analysis of variance, and multiple comparison tests (unpaired students ‘-t’ test) were performed when significant differences were found between the subgroups. In the case of skewed distribution, a non-parametric Mann Whitney test was used for comparing data between two groups, and for more than two groups, a non-parametric Kruskal Wallis test was used. Statistical significance of categorical variables was determined by the chi-square test. p<0.05 was taken as a level of statistical significance.

## Results

A total of 400 participants were included in the study, with 350 cases and 50 controls. The cases were allocated to seven groups according to the rhythm (AF/NSR), valvular pathology (MS/MR/MS with MR), and presence or absence of left atrium/left atrial appendage (LA/LAA) clot (Table 1). Demographic and echocardiographic data of the study population was as described in Table 2. Out of 400 subjects, 48% (n=192) were males while 52% (n=208) were females. The sex ratio was similar in all study subgroups.

**Table 2:** Patient characteristics and echocardiographic parameters of the study population MS- mitral stenosis; MR- mitral valve regurgitation; NSR- normal sinus rhythm; AF- atrial fibrillation; LA- left atrium; LAA- left atrial appendage, SEC- spontaneous echo contrast

	Age (years) (mean ±SD) (Range)	Male N (%)	Left atrium diameter (mm) (mean ±SD)	Left atrium SEC^#^(N) (%)	
					MS with NSR	30.28± 5.47 (18-42)	21 (42%)	45.9±5.8	9(18%)	
MS with AF	33.8±9.52 (19-65)	23 (46%)	49±11.8	24(48%)	
MR with NSR	30.62±7.63 (18-58)	23 (46%)	47.9±9.13	0 (0%)	
MR with AF	32.44±5.57 (23-54)	32 (64%)	51.4±12	0 (0%)	
MS with MR with NSR	33.48± 7.7 (22-60)	21 (42%)	47.9±13.14	0 (0%)	
MS with MR with AF	32.56 ± 6.83 (19-58)	30 (60%)	52.6±8.27	0 (0%)	
MS and/or MR with clot in LA/LAA	34.42±8.06 (22-60)	20 (40%)	52.2±8.21	32 (64%)	
Control	31.4±4.38 (22-47)	22 (44%)	28±2.8	0 (0%)	
P-value (as compared with control)	0.02	0.13	<0.001	0	

The mean (± SD) age of study subjects was 32.32±7.30 years (range 30-65 years). The mean age was similar in all study subgroups except in patients with the LA/LAA clot group. Age in the LA/LAA clot group was significantly higher compared to the control group (34.42 ± 8.06 years vs 31.4 ± 4.38 years; p = 0.02).

D-dimer levels showed skewed distribution in the total study population as well as in study subgroups. Plasma D-dimer levels of various study subgroups are shown in Table 3. The median level of D-dimer in the control group was 0.31 µg/ml (range: 0.08-0.53). The highest median level was found in the LA/LAA clot group (1.0 µg/ml, range: 0.14-9.57). This was significantly higher compared to the control group (p = 0.03). Patients with MS (MS with AF and MS with NSR) had significantly higher plasma D-dimer levels than controls (p <0.001). Regardless of atrial rhythm, patients in the MR subgroups (MS with MR and only MR subgroups) had normal levels of D-dimer that were similar to healthy controls in sinus rhythm (p = non-significant).

**Table 3:** Plasma D-dimer levels in study subgroups MS- mitral stenosis; MR- mitral valve regurgitation; NSR- normal sinus rhythm; AF- atrial fibrillation; LA- left atrium; LAA- left atrial appendage

GROUP	Mean (µg/ml)	Standard Deviation (µg/ml)	Median (µg/ml)	Range (µg/ml)	p-value
MS with NSR	0.85	0.95	0.62	0 - 5.95	<0.001
MS with AF	1.33	1.73	0.65	0.05 - 8.48	<0.001
MR with NSR	0.29	0.17	0.31	0.08 - 1	0.53
MR with AF	0.32	0.23	0.30	0.07 - 1.17	0.23
MS with MR with NSR	0.38	0.34	0.30	0.05 - 1.66	0.14
MS with MR with AF	0.39	0.21	0.32	0.04 - 0.8	0.34
MS and/or MR with clot in LA/LAA	1.71	1.74	1	0.14 - 9.57	0.03
CONTROL	0.31	0.11	0.31	0.08- 0.53	0

**Figure 1:**
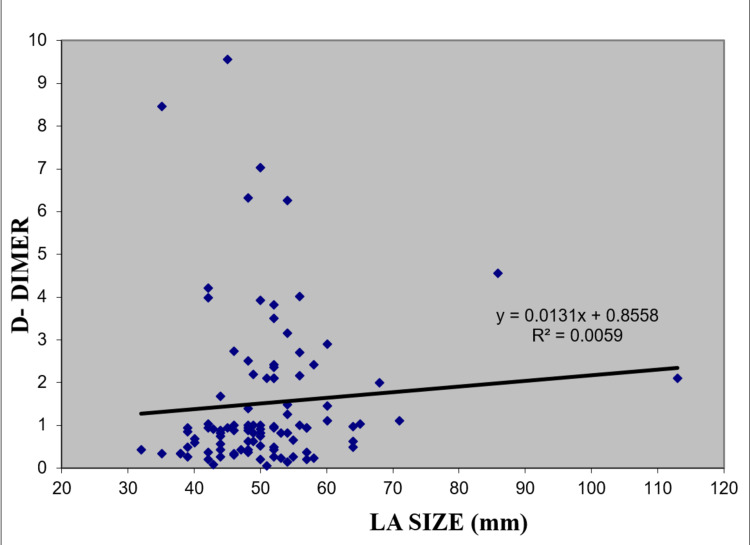
Scatter diagram for the relationship of LA size to D-dimer in MS with NSR, MS with AF, and MS and/or MR with LA/LAA-clot subgroup LA- Left atrium; MS- mitral stenosis; NSR- normal sinus rhythm; AF- atrial fibrillation; MR- mitral valve regurgitation, LAA- left atrial appendage

When the relation of LA diameter and plasma D-dimer was studied, it was not significant for any of the individual study subgroups. However, combined analysis of MS subgroups (MS with NSR, MS with AF, and MS and/or MR with LA/LAA clot) showed a linear relationship between LA diameter and plasma D-dimer levels (Spearman correlation coefficient=0.232 with a p-value of 0.02) (Figure 1).

## Discussion

The risk of thromboembolism is increased 17-fold in patients with chronic atrial fibrillation associated with rheumatic mitral valve disease [26]. Both AF and MS cause stagnation of blood in the left atrium and promote thrombus formation. Unsurprisingly, evidence of valvular heart disease occurring concomitantly with AF merits anticoagulation therapy. Severe MR creates a high-flow wash-out jet, which decreases coagulation activity and thrombus formation and inhibits thrombogenesis in the left atrium [6,8].

In the present study, we hypothesized that significant mitral regurgitation is protective against LA stasis and clot formation in rheumatic mitral valve disease. An indirect assessment of the LA procoagulant milieu was made by systemic D-dimer, which is a well-known marker of increased coagulation and fibrinolytic activity in the body. Levels were significantly raised in patients with mitral stenosis (whether in AF or normal sinus rhythm) and the highest levels were found in the presence of LA/LAA clots. Regardless of atrial rhythm, patients with MR had normal levels of D-dimer that were comparable to healthy controls in sinus rhythm. Even in patients with MS who have concomitant severe MR, levels were similar to controls. Thus, MR is protective against coagulation tendency predisposed by MS and AF.

Many small-scale studies showed that levels of these procoagulant markers are increased in the presence of mitral stenosis and/or atrial fibrillation [1,27]. In a study of 36 patients, Roldan et al. showed that D-dimer levels in patients with AF (both rheumatic and non-rheumatic) were substantially higher than in controls (549.38 ± 311.16 ng/ml versus 12.3 ± 3.7 ng/ml, p 0.001) [1]. They postulated that AF induces a state of enhanced intravascular clotting, resulting in significantly raised D-dimer levels in the blood. Hayashi et al. also showed that D-dimer levels were raised in all patients with mitral stenosis [27]. When compared to the control group, median values were significantly higher (Thrombus present: 378 ng/ml; Thrombus absent: 93 ng/ml; Normal controls: 64 ng/ml) (p=0.01 in both cases).

The present study shows that D-dimer levels are significantly higher in rheumatic mitral stenosis. Though the presence of AF in these patients is associated with higher levels compared to normal sinus rhythm, this association does not qualify for statistical significance.

High D-dimer levels also correlate with the presence of spontaneous echo contrast and LA clot. Heppell et al. found that raised median D-dimer levels were predictive of an LA clot (median value 479 ng/ml [interquartile range: 334 -738] in LA clot group vs 298 ng/ml [IQR: 175 -502] in the absence of a clot; p = 0.004) [28]. Somlói et al. showed that normal D-dimer had a very high negative predictive value for an LA clot [23]. A normal plasma D-dimer level reliably excludes an atrial thrombus in patients with AF, with a negative predictive value of 98%. In our study also, the presence of an LA clot was associated with the highest levels of D-dimer that were higher than MS subgroups without an LA clot irrespective of the atrial rhythm.

There was no relationship of LA size with D-dimer level in our study, which suggested that the increase in D-dimer levels in these patients is related to stasis of blood due to slow flow rather than an increase in LA size.

The most important finding in the present study is that patients with severe mitral regurgitation have D-dimer levels similar to controls regardless of atrial rhythm. Both the only MR and MS with MR subgroups have levels similar to controls. Even on intergroup comparisons, levels are similar amongst all subgroups with significant MR (Only MR and MS with MR subgroups) regardless of atrial rhythm. Thus, severe MR has an inverse relationship to D-dimer levels. Few echocardiographic series have also revealed an inverse relationship between the severity of MR and left atrial thrombus and/or SEC prevalence [6-9]. For example, Movsowitz et al. considered severe MR to be an independent negative predictor for left atrial SEC and/or thrombus [6].

To our knowledge, in the only study to date, Cevik et al. [21] also studied plasma D-dimer levels as a procoagulant marker to investigate the impact of rheumatic MR on systemic coagulation activity. Patients with MS with AF, MS with NSR, and nonvalvular AF had significantly higher D-dimer levels than healthy controls (p <0.01) while D- dimer level was normal in patients with MR. However, the study had limited statistical power due to the small sample size (n=98).

The results of the present study suggest that significant mitral regurgitation decreases coagulation activity and thrombus formation in the left atrium of patients with atrial fibrillation and mitral stenosis. Current practice guidelines recommend that all patients with chronic AF and rheumatic mitral valve disease receive oral anticoagulants (Class I Recommendation, Level of Evidence: A) in a dose adjusted to an international normalized ratio (INR) of 2.0 to 3.0 unless contraindicated [25]. Our findings suggest that those patients with concomitant severe MR may require a less intensive regimen, such as a lower INR goal or aspirin-only treatment, and a reconsideration of these treatment recommendations is encouraged. We also suggest that plasma D-dimer levels have clinical utility in the assessment of thromboembolic risk in patients with AF or MS.

The results of the present study establish that plasma D-dimer is a sensitive marker of increased coagulation activity in MS and AF. Concomitant severe MR is protective against the procoagulant state as reflected by a normal plasma D-dimer level. Thus, a low D-dimer with severe MR has a significant clinical value in deciding the need for anticoagulation therapy. Since long-term anticoagulation is fraught with risk, and the ability to safely avoid such risks in this population would be very helpful.

Study limitation

Trans-esophageal echocardiography was not done in our study, which would have better assessed LA/LAA for the presence of clots in patients. Moreover, other markers of thrombogenicity like fibrinogen were not studied, which would have added more evidence to the study.

## Conclusions

Mitral stenosis and atrial fibrillation increase the risk of thromboembolism while, in contrast, severe MR reduces plasma D-dimer concentrations to control levels, a result that may reflect the protective effect of severe MR against left atrial thrombosis and systemic embolization. Since long-term anticoagulation is fraught with risk, and the ability to safely avoid such risks in these populations would be very useful. Thus, patients with severe MR might require less aggressive anticoagulation regimens.
